# Biochemical and biomolecular effects induced by a static magnetic field in *Saccharomyces cerevisiae*: Evidence for oxidative stress

**DOI:** 10.1371/journal.pone.0209843

**Published:** 2019-01-04

**Authors:** Ameni Kthiri, Slah Hidouri, Tahri Wiem, Roua Jeridi, David Sheehan, Ahmed Landouls

**Affiliations:** 1 Laboratory of Biochemistry and Molecular Biology, Carthage University, Faculty of Sciences of Bizerte, Zarzouna, Bizerte, Tunisia; 2 Environmental Research Institute and School of Biochemistry and Cell Biology, University College Cork, Western Gateway Building, Western Road, Cork, Ireland; 3 Dept of Chemistry, College of Arts and Sciences, Khalifa University of Science and Technology, Abu Dhabi, United Arab Emirates; Aligarh Muslim University, INDIA

## Abstract

Exposure to static magnetic fields (SMF) can cause changes in microorganism metabolism altering key subcellular functions. The purpose of this study was to investigate whether an applied SMF could induce biological effects on growth of *Saccharomyces cerevisiae*, and then to probe biochemical and bio-molecular responses. We found a decrease in growth and viability under SMF (250mT) after 6h with a significant decrease in colony forming units followed by an increase between 6 h and 9 h. Moreover, measurements of antioxidant enzyme activities (catalase, superoxide dismutase, glutathione peroxidase) demonstrated a particular profile suggesting oxidative stress. For instance, SOD and catalase activities increased in magnetized cultures after 9 h compared with unexposed samples. However, SMF exposure caused a decrease in glutathione peroxidase activity. Finally, SMF caused an increase in MDA levels as well as the content of protein carbonyl groups after 6 and 9 h of exposure.

## Introduction

In the near future the terrestrial atmosphere will be permeated by an increasing variety of man-made magnetic fields such as low frequency pulse magnetic fields in addition to the Earth’s own magnetic field [1]. Key anthropogenic sources of electromagnetic fields are high voltage power lines and electronic applications such as magnetic resonance imaging [2]. In addition, many devices work with weak magnetic fields such as cellular phones, computers and magnet-associated engines. There is concern about the health and other risks such radiation may pose to humans and other organisms. Therefore, systematic investigation of exposure effects in model biological systems can contribute to risk assessment. Currently, interaction mechanisms are unclear and there is considerable variation in experimental design and endpoints chosen for study [3]. Simple organisms such as microorganisms (yeast or bacteria) are convenient for controlled experimentation [4]. Higher animals such as mice [5, 6] are used in order to simulate effects on humans [7, 8].

Effects amenable to study in microorganisms include their viability and proliferation [9], morphology [10], apoptosis [11], genotoxicity [12], metabolic activity [13], activity of enzymes [14, 15], gene expression and transport of ions into the cell [16]. Amongst microorganisms studied in this way, brewer’s yeast, *Saccharomyces cerevisiae*, is an excellent eukaryotic model. This yeast can also be used to investigate the effects of SMF on cell growth and stress effects associated with continuous or chronic exposure. Firstly, due to the availability of a well-characterized and complete genome. Secondly, yeast cells share many basic cellular processes with other eukaryotes including humans [17]. Previous SMF studies with *S*. *cerevisiae* include those of Motta *et al*. [18], Nakayashiki *et al*. [19] and Egami *et al*. [20].

It seems that each microorganism has a specific response profile against SMF exposure. For example, Nakasono *et al*. [21] did not report any changes in protein synthesis or cell cycle after exposure of yeast to SMF for 24 h. Similar observations were made in *Escherichia coli* [22]. However, when SMF intensity became greater, oxidative stress was triggered. Indeed, Friedl *et al*. [23] mention that, when a yeast strain was subjected to physical stressors such as SMF, oxidative stress or cytotoxic factors induced cell damage. Wild type yeasts are relatively resistant to external stressors due to efficient repair mechanisms which mitigate against damage induced by physical or chemical agents even at high doses. In the current study we explored biochemical and biomolecular profiles in yeast exposed for varying times to SMF at 250 mT. Various parameters were tested in order to better understand the behavior of the yeast under SMF.

## Materials and methods

### Yeast strain and culture medium

Experiments were performed with a wild type yeast strain *S*. *cerevisiae* BY4741 (genotype:*MATa his3*Δ*1 leu2*Δ*0 met15*Δ*0 ura3*Δ*0*) supplied by Euroscaf collection. Yeast cells were grown in a medium of YPD broth (1% Bactoyeast extract, 2% Bacto-peptone, 2% glucose) with or without 2% Bacto-agar).

### Static magnetic field exposure

For SMF a montage was assembled composed of a pair of cylindrical coils spaced by 11 cm (each coil: diameter, 20 cm; length, 13 cm; Beaudouin, France) supplied by four generators delivering a direct current. Coils were water-cooled and the temperature inside the coils was maintained at room temperature. An Erlenmeyer-shaped glass double phial was used as a sample holder. Exposures were performed with a magnetic induction of about 250 mT in the middle of the double phial. The temperature was maintained at 30°C inside the glass double phial by water circulation, using an incubator system composed of pump and resistance.

### Growth and viability measurements

Fresh yeast cultures were used for all experiments. Pre-cultures were grown overnight at 30°C in 10 mL of culture medium in 18-mm diameter tubes. Then, they were diluted in 50 mL of sterile nutrient broth in an Erlenmeyer-shaped glass double phial to an initial concentration corresponding to OD_600_ = 0.1. Control cultures were maintained under the same conditions as exposed ones except that the magnetic field was turned off. For cell proliferation analysis, diluted yeast cultures were exposed to magnetic field for 9 h and the OD measured each hour. A1 mL aliquot was withdrawn under sterile conditions from both the control and exposed cultures for examination by light spectrometry at 600 nm. The viable cell numbers were expressed as the log_10_ of the surviving fraction given in number of colony forming units (CFU) per mL (N) divided by the initial number of CFU per mL (N_0_). The logarithms (N/N_0_) were plotted against time. The number of CFU was determined 3, 6, and 9 h after starting cultivation. Spectrophotometric and CFU measurements were carried out for six independent exposure experiments.

### Preparation of cell-free extracts for biochemical and biomolecular assays

Exposed or control cells from 6 and 9 h cultivations were collected by centrifugation at room temperature (5 min, 7000 *g*) and washed with 50mM potassium phosphate (K-phosphate) buffer (pH 7.0). The yeast pellets were re-suspended in lysis buffer (50 mM K-phosphate buffer with 1 mM of phenylmethylsulfonyl chloride and 0.5 mM EDTA). The cell suspensions were vortexed for 15 cycles of 1 min of vortexing with 1 volume of glass beads (0.5 mm) followed by 1 min of cooling on ice. Cell debris was removed by centrifugation for 10 min at 15,000 *g*. The cell extract was kept in ice for immediate use [24]. Protein concentration from each cell free sample extract was quantified by the method of Bradford [25] using bovine serum albumin (BSA) as a standard.

### Enzyme activities

Biochemical measurements of glutathione peroxidase (GPX) and the antioxidant enzymes superoxide dismutase (SOD) and catalase (CAT) were conducted using a spectrophotometer/microplate reader and activities were measured separately. Each enzymatic activity was determined in triplicate for each sample.

SOD activity was measured as described by Ewing and Janer [26]. The reaction mixture contained 62.5 mM sodium phosphate buffer (pH 7.4), 0.125 mM EDTA, 83.3 mM NBT, 150 mM NADH and 16.5 mM PMS. Reduction of NBT was monitored at 25°C as an absorbance increase at 560 nm (every 20 or 30 s) for 5 min. One unit of SOD activity is defined as that amount of enzyme causing 50% inhibition of NBT [26].

CAT activity was measured by monitoring the disappearance of hydrogen peroxide at 240 nm using the extinction coefficient of hydrogen peroxide (39.4 mM^-1^cm^-1^). One unit of catalase activity was defined as the μmoles of hydrogen peroxide decomposed per min [27]

GPX activity was determined using the method of Paglia and Valentine [28] in which GPX activity was coupled with the oxidation of NADPH by glutathione reductase. The reaction mixture comprised 50 mM potassium phosphate buffer (pH:7), 1mM EDTA, 1 mM NaN_3_, 0.2 mM NADPH, 1 mM glutathione and 1 U/mL of glutathione reductase. The activity of GPX was assayed by the subsequent oxidation of NADPH at 340 nm using an extinction coefficient of 6.22 mM^-1^ cm^-1^. The results were expressed as unit per mg of protein.

### Biomolecular characterization

Measurement of protein carbonyls: The content of carbonyl groups in proteins was measured by determining the amount of 2,4-dinitrophenylhydrazone formed upon reaction with DNPH. The cell-free extract (>1.5 mg protein) placed in glass tubes were treated with 10 mM DNPH in 2 M HCl at room temperature for 1 h of incubation in the dark and vortexed every 15 min. Blanks contained 2 M HCl without DNPH. Proteins were precipitated by addition of trichloroacetic acid (TCA) up to final concentration of 10%, then centrifuged at 4000 *g* for 10 min at 4°C, and washed three times with 1 mL ethanol: ethyl acetate (1:1). The final pellets were dissolved in 6 M guanidine hydrochloride in 5% (v/v) phosphoric acid. Carbonyl content was calculated from the absorbance maximum of 2, 4-dinitrophenylhydrazone measured at 370 nm using an extinction coefficient of (22 mM^-1^cm^-1^) [29]. The results are expressed in nanomoles per milligram of protein.

Lipid peroxidation: Malondialdehyde (MDA) was used as an index of lipid peroxidation. Quantification of MDA is based on its reactivity to thiobarbituric acid (TBA). MDA was determined by the modified method of Steels *et al*. [30]. Briefly, dry yeast cells (50 mg) cooled on ice were harvested, centrifuged and washed with distilled water. The pellets were re-suspended in 0.5 mL of 10% TCA (w/v) and 1.5 g of glass beads were added. The samples were disrupted by 6 cycles of 20 s agitation on vortex mixer followed by 20 s on ice. Following centrifugation, the supernatant was mixed with 0.1 mL of EDTA (0.1 M) and 0.6 mL of TBA (1%) in NaOH (0.05 M). The reaction mixture was incubated at 95°C for 15 min and the absorbance was measured at 532 nm using the molar extinction coefficient for MDA (1.56×10^5^ M^-1^cm^-1^) mM^-1^.

### Statistical analysis

The results were plotted and statistical analyses were performed using data analysis software system SPSS PASW statistics 18. The results are expressed as the mean values ± standard deviations.

The significance of difference between results were evaluated using one-way analysis of variance (ANOVA) followed by Tukey’s *post hoc* HSD test because this test compares all treated groups including controls defined by a single factor. A P value of 0.05 or less was considered significant.

## Results

### Effects of SMF on the survival of *Saccharomyces cerevisae*

**Fig 1:**
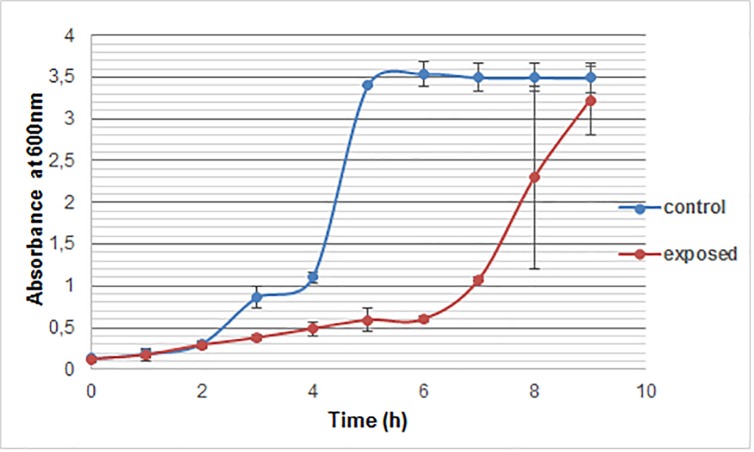
*S*. *cerevisiae* growth as a function of exposure time to SMF (250 mT). Values are expressed as means ± SD of at least six different spectrophotometric measurements. Control cells are shown in blue while SMF-exposed cells are shown in yellow.

The growth of *S*. *cerevisae* is characterized by a model curve with, usually, a latency period starting from the beginning of the culture for a few hours, then an exponential phase and finally a stationary phase. Each phase varies according the condition of the culture. As shown (**Fig 1**), no proliferation differences were observed between exposed and control cells between 1 and 3 h, the values of OD remain, respectively, the same (0.3 and 0.29 U OD at 3 h). However, SMF exposure induced a decrease in cell growth at 6 h compared to unexposed cells. The values of OD are, respectively, 0.59 and 3.5 for exposed cells and the control.

**Fig 2:**
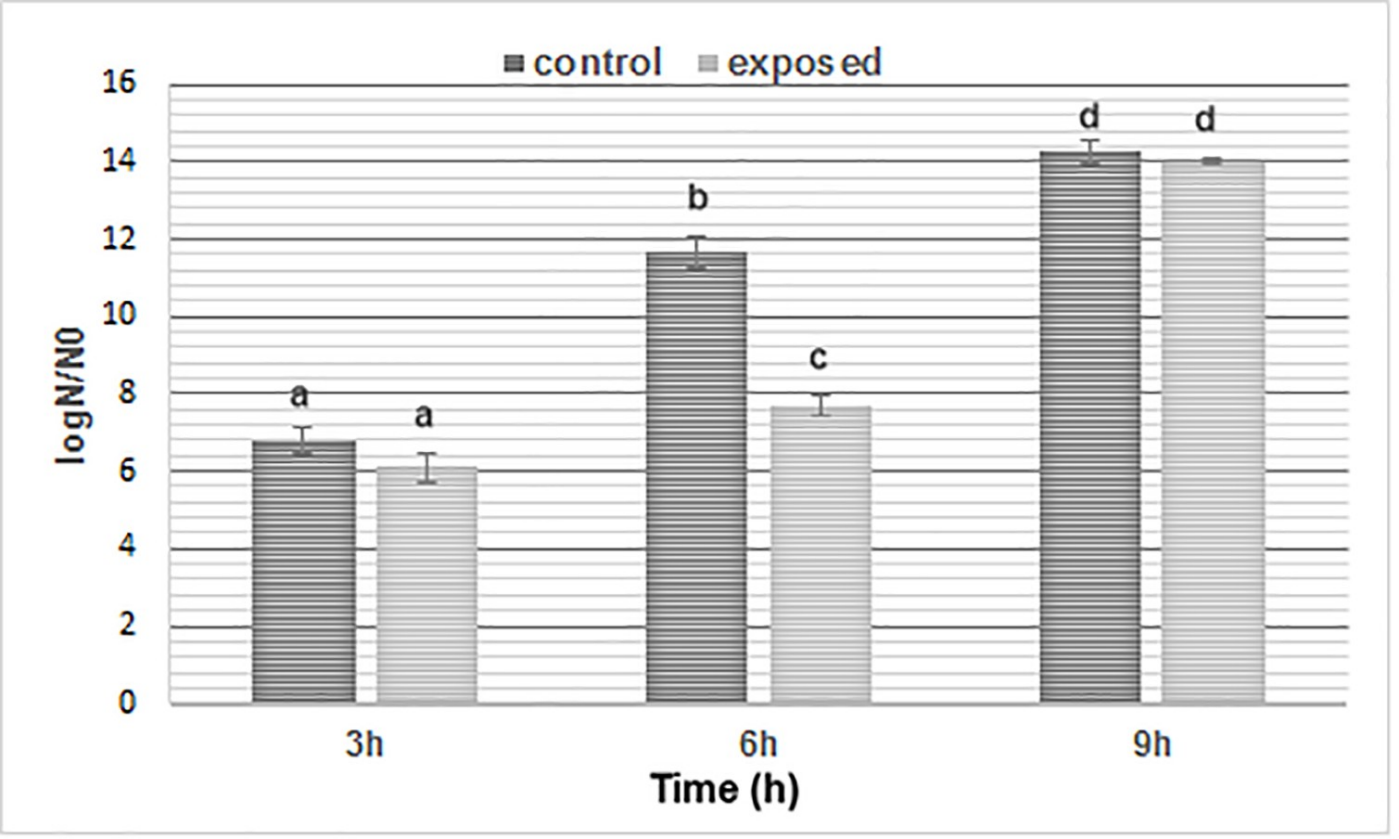
Viability changes in *Saccharomyces cerevisiae* during SMF exposure. The data are plotted as log_10_ of the ratio of the number of surviving colony-forming units (CFU) per ml divided by the initial number of CFU per ml at time zero. The logarithms are plotted against time and the values are expressed as means ± SD of six different CFU measurements. Significant differences are indicated by different letters vs. control samples (b: p < 0.05, c: p < 0.01 and d: p < 0.001).

To better detect the effect of SMF on the dynamics of growth, numbers of viable cells in the exposed and unexposed yeast were compared after 3, 6 and 9 h incubation. The results (**Fig 2**) reveal that there is no significant difference (p > 0.05) between exposed and control samples at 3 h, whereas, at 9 h, the SMF exposure induced a decrease of viable cells (p < 0.05) followed by an increase of CFU in exposed cells at 9 h which was almost comparable with control. On the basis of this observation 6 and 9 h of exposure to SMF were selected to study biochemical and bio-molecular effects on *S*. *Cerevisiae* under SMF.

**Fig 3:**
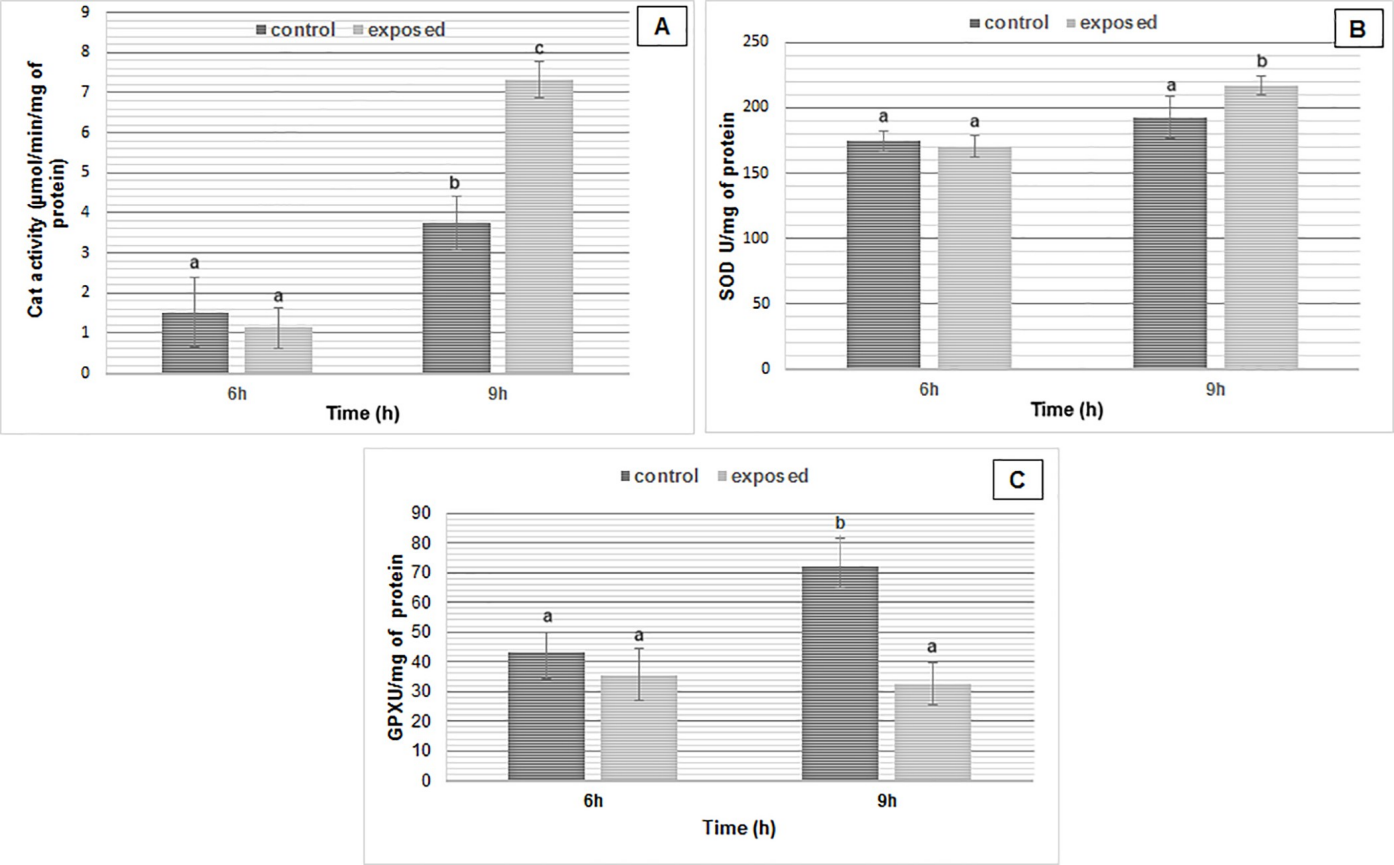
Activities of antioxidant enzymes in wild-type cells of *S*. *cerevisiae* exposed to SMF (250 mT). (A) CAT, (B) SOD, and (C) GPX. The results are expressed as the means of at least three independent determinations ± SD. Significant differences are indicated by different letters (a: p < 0.05, b: p < 0.01 and c: p < 0.001).

CAT activity (**Fig 3A**) was initially lower (1.514 μmol/min/mg) in cells exposed for 6 h to SMF. However, after 9 h of exposure, a significant almost two-fold increase of CAT activity was evident (P < 0.05). Conversely, a different result was observed for SOD activity (**Fig 3B**). Cells exposed to SMF for 6 h showed similar SOD activity levels with only a modest increase at 9 h exposure. GPX activity showed no significant effect in cells exposed for 6 h (P > 0.05) (**Fig 3C**). In contrast, GPX activity was strongly reduced after 9 h of exposure which decreased approximately 2-fold.

In conclusion, we observed that activities of both catalase and SOD were higher in exposed cells than control, with the most pronounced increase being noted after 9 h of SMF exposure. On the other hand, GPX activity showed a different response pattern.

### Effect of SMF on protein carbonyl content and lipid peroxidation

**Fig 4:**
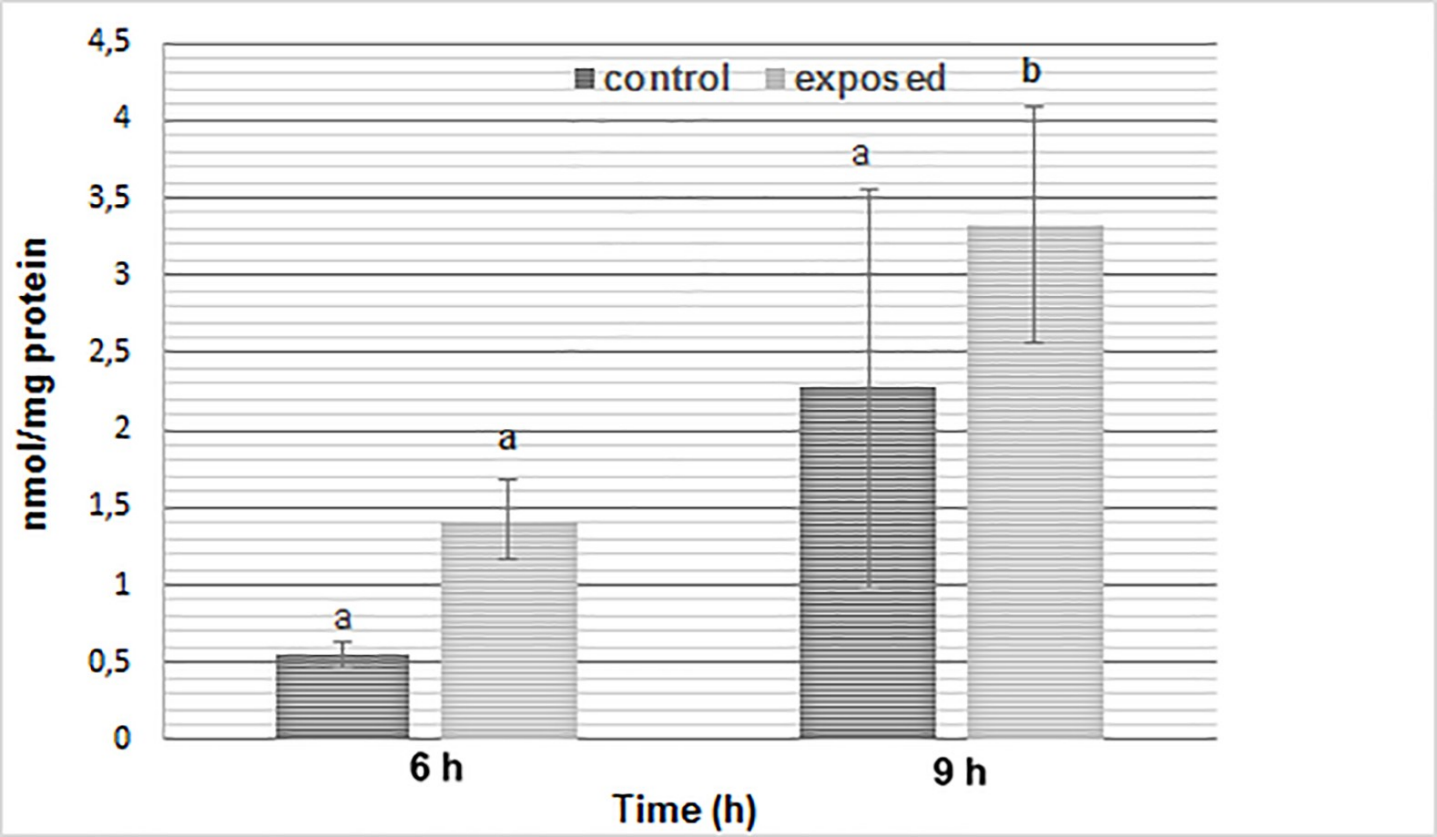
The level of protein carbonyl groups of wild-type cells of *S*. *cerevisiae* before and after exposure to SMF (250 mT). The results are given as means of at least three independent determinations ± SD. Significant differences are indicated by different letters (a: p < 0.05 and b: p < 0.01).

Protein carbonyl levels of *S*. *cerevisiae* after 6 h of SMF exposure were not significantly different to control cells (P < 0.05). However, after 9 h exposure, protein carbonyl level increased significantly (**Fig 4**).

**Fig 5:**
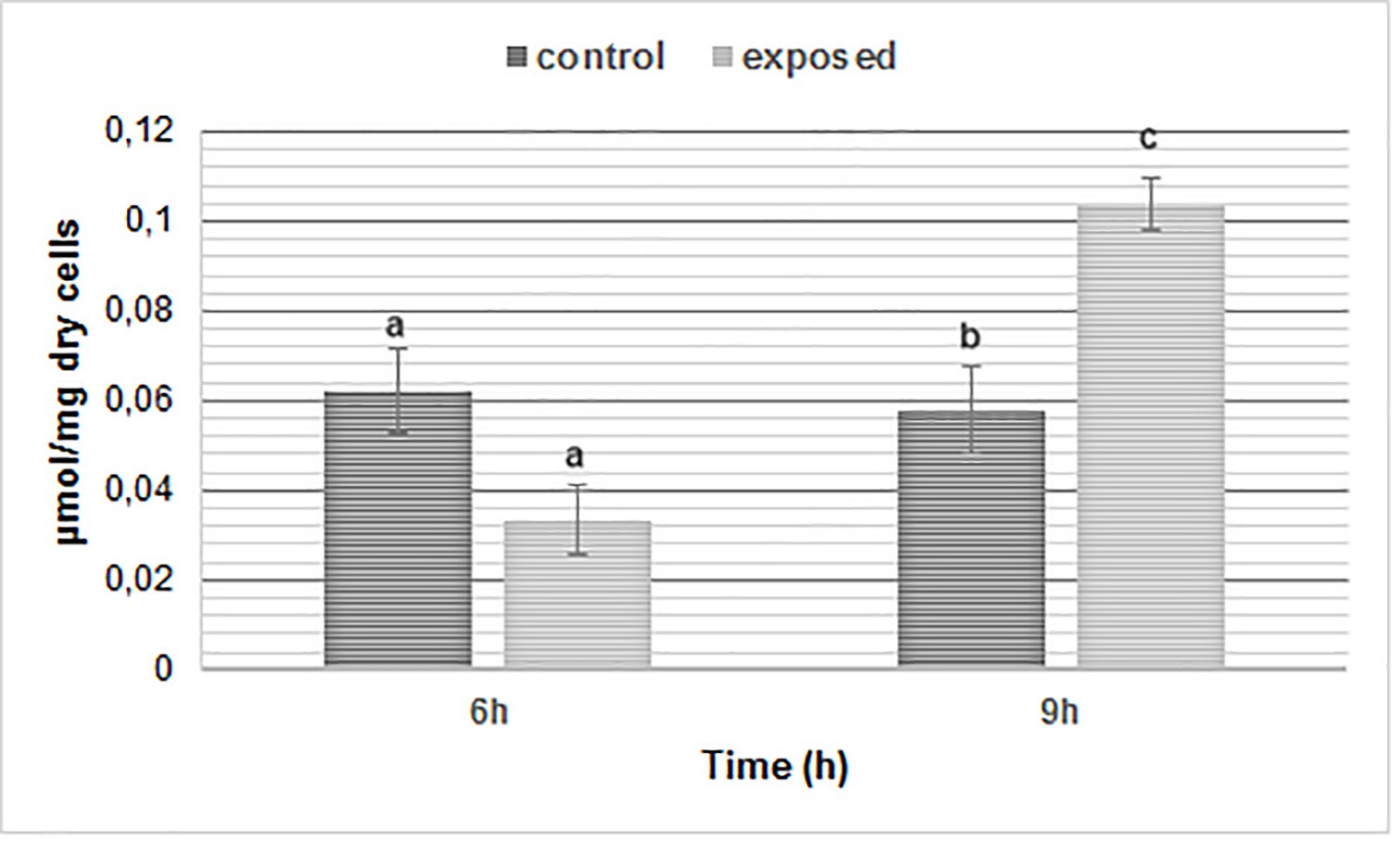
Lipid peroxidation of wild-type cells of *S*. *cerevisiae* before and after exposure to SMF (250 mT). The results are the means of at least three independent determinations ± SD. Significant differences are indicated by different letters (a: p < 0.05, b: p < 0.01 and c: p < 0.001).

SMF exposure for 6 h caused approximately 50% decrease in MDA levels. A significant increase of MDA concentration (P < 0.05) was observed in SMF-exposed cells at 9 h (**Fig 5**).

## Discussion

The present study aimed at exploring effects of SMF (250 mT) on *S*. *cerevisiae* growth and viability as well as analysis of some molecular mechanisms in response to SMF. Whilst several studies have previously exposed yeast cells to varying strengths and durations of both static and alternative MFs, the results have often been contradictory. For example, it was shown that the number of viable yeast cells and their cell growth rate decreased after exposure to a homogeneous SMF at 10 mT [31]. However, Strašak *et al*. [32] reported that, at temperatures 24–26°C, a cylindrical coil of 10 mT and a frequency of 50 Hz significantly decreased the growth of *S*. *cerevisiae* [32]. Related to that it has been demonstrated that the growth rate of yeast strains exposed to SMF with strength in the range 0–10 T, were suppressed by the SMF up to 5 T [33]. In addition, a study carried out by Binninger and Ungvichain [34] demonstrated that *S*. *cerevisiae* gene expression was altered by exposure to 20 μT 60 Hz magnetic fields over a period of 15 cell generations. Nevertheless, it was demonstrated that *S*. *cerevisiae* exposed to low intensities and frequencies for a longer time to a static and 50 Hz sinusoidal magnetic field at 0.35 and 2.45 mT with exposure times of 24 and 72 h failed to alter the growth of WS8105-1C yeast strains in comparison with unexposed cells [35]. Even at 159 mT, growth of *S*. *cerevisiae*, spread on agar plates were not altered [36]. Yet, in the present study, growth was shifted especially during the latency phase suggesting the yeast requires some time to manage the change in its environment.

Coupled with CFU counting, growth in liquid medium showed suppressed behavior after 6 h exposure. However, with longer exposure of 9 h, growth was unchanged which is consistent with a study by Iwasaka *et al*. [33]. These workers showed that the rate of yeast proliferation decreased after exposure to magnetic fields (9–14 T) compared to unexposed cells. It seems that cells required approximately 7 h for lag-phase before starting the exponential phase.

Several systems are used by most living organisms to protect themselves from oxidative stress including enzyme and non-enzyme molecules. Antioxidant enzymes are major elements of such systems which include SOD, CAT, and GPX [37]. Our results showed that exposure to SMF (250 mT) significantly influenced some of these biochemical markers. SOD and CAT activities increased significantly in cells after 9 h SMF exposure. SOD is primarily important for ROS detoxification and is a key antioxidant enzyme catalyzing dismutation of two superoxide radicals to hydrogen peroxide and oxygen [38]. Increased SOD activity *in S*. *cerevisiae* implies that a moderate SMF (250 mT) can be a physical agent to stimulate the production of ROS. Furthermore, an increase in SOD activity could be a signal for the activation of stress-response, defense pathways and could constitute an adaptive response to SMF in *S*. *cerevisiae*.

Following exposure to SMF, SOD activity promotes the accumulation of hydrogen peroxide via dismutation of the superoxide anion. Elimination of this molecule is a role for CAT activity. Respecting the data shown here, an increase in CAT activity on 9 h exposure to SMF may be related to induction of SOD activity triggering production of hydrogen peroxide. This, in turn, may lead to activation of CAT as a classical route to overcome oxidative stress. Cells possess other enzymatic antioxidants such as GPXx and TRX peroxidases/peroxiredoxins (PRX), which use electron-donating cysteine thiol groups to catalyze hydroperoxide reduction [39]. Our data demonstrated a significant decrease of GPX activity in yeast exposed to SMF (250 mT). It seems that this antioxidant enzyme is more sensitive toward SMF than either CATor SOD enzymes. A decrease in GPX activity could be explained by direct inhibition of the enzyme by their binding with oxidative molecules produced during exposure to the magnetic field [6] or, potentially, due to accumulation of hydrogen-peroxide leading to lipid peroxidation [40]

There are relatively few reports on the effect of SMFs on antioxidative defense parameters in *S*. *cerevisiae*. Recently in our laboratory a study conducted on prokaryotic cells (*Pseudomonas aeruginosa*) revealed a protective role for SOD against SMF exposure (200 mT, 8 h) in wild type and mutant strains, and increased levels of antioxidant activities such as SOD, CAT and GPX [41]. On the other hand, in studies carried out with higher organisms, Ghodbane *et al*. [42] noted decreased GPXactivity in muscles and kidneys, and an increase of SOD activity in the liver after exposure to SMF (128 mT) [42]. However, Amara *et al*. [43] showed decreased activities of CAT, CuZn-SOD, and GPX in rat livers and kidneys. In addition, Kerzeja *et a*l. [44] and Kula *et al*. [45], demonstrated SMF with 0.49 T and 128 mT intensities did not affect SOD, GPX or CAT activity in human and mouse fibroblasts.

Antioxidant enzymes studied here are part of antioxidant defense mechanisms developed by *S*. *cerevisiae* to protect against ROS. Accumulation of cellular ROS inevitably results in oxidative damage to important cell biomolecules such as proteins, DNA, and lipids. At this level, the study probed whether a moderate SMF (250 mT) could induce damage to proteins and lipids. Oxidative damage to lipid molecules generally involves lipid peroxidation, an autocatalytic process initiated by the oxidation of polyunsaturated fatty acids caused by ROS. Peroxidation of unsaturated fatty acids leads to formation of products such as MDA or 4-hydroxynonenal (4-HNE) [46, 47, 48, 49]. After 9 h of exposure we found that MDA concentration was two-fold higher than control, which is similar to the observations of Amara et al. [50]. These workers showed that exposure to SMF (128 mT; 1 h/day for 30 days) could induce an increase in MDA concentration in rat testis. In contrast, Kabuto *et al*. [5] demonstrated that exposure to a SMF of about 1.5–300 mT had no effect on MDA accumulation in brain homogenates which illustrates diverse biological responses to SMF in differing biological contexts.

Protein carbonylation is an early marker for protein oxidation resulting from covalent modification of specific protein side-chains (lysine, arginine, proline and histidine) [51, 52]. Carbonyl groups may also be formed in proteins by Michael addition reactions of 4-HNE or by modifications with reducing sugars and can induce conformational modifications, impaired function, and proteolysis [53].

Interestingly, our study showed a significant elevation in protein carbonyls after 9 h of SMF exposure. This demonstrates that SMF produces ROS and promotes chemical and conformational modification of yeast proteins. Many previous studies agree with these observations and showed that glycolytic enzymes such as glyceraldehyde-3- phosphate dehydrogenase and phosphoglyceromutase, mitochondrial enzymes and Cu,Zn-SOD could be major targets for oxidation and inactivation [54, 55].

## Conclusion

SMF is reported to have various effects on microorganisms. This study confirms that *S*. *cerevisiae* responds with a large decrease in latency phase which was considered as an adaptive phase of the growth kinetic. Oxidative stress is suggested by an increase in CAT and SOD activities after 9 h of exposure with a decrease of GPX activity along with an increase in MDA and carbonylated proteins in response to SMF. These biochemical markers may be very informative in probing deeper into SMF effects on yeast cells.
